# Pre-Operative Embolisation of Musculoskeletal Tumours - A Single Centre Experience

**DOI:** 10.5704/MOJ.2003.007

**Published:** 2020-03

**Authors:** SJ Wong, T Urlings, C Seng, S Leong, BS Tan, MH Tan

**Affiliations:** 1 Department of Orthopaedic Surgery, Singapore General Hospital, Singapore; 2 Department of Radiology, Haaglanden Medical Centre, The Hague, The Netherlands; 3 Department of Vascular and Interventional Radiology (DVIR), Singapore General Hospital, Singapore

**Keywords:** pre-operative embolisation, tumours, blood loss, devascularisation, interventional radiology

## Abstract

**Introduction:**

The management of musculoskeletal tumours is complex and requires a multi-disciplinary approach. Preoperative embolisation can be often employed to reduce intra-operative blood loss and complication rates from surgery. We report our experience with the safety, technical success and efficacy of pre-operative embolisation in musculoskeletal tumours.

**Materials and Methods:**

Thirteen consecutive patients who underwent pre-operative embolisation of a musculoskeletal tumour followed by surgical intervention at our institution from May 2012 to January 2016 were enrolled into the study. Patient demographics, tumour characteristics, embolisation techniques and type of surgery were recorded. Technical success of embolisation, amount of blood loss during surgery and transfusion requirements were estimated.

**Results:**

There were five female and eight male patients who underwent pre-operative embolisation during the study period. The age ranged between 16 to 68 years, and the median age was 54. Technical success was achieved in all patients. Mean intra-operative blood loss was 1403ml, with a range of 150ml to 6900ml. Eight patients (62%) required intra-operative blood products of packed red blood cells and fresh frozen plasma. No major complications occurred during embolisation.

**Conclusion:**

Pre-operative trans-arterial embolisation is feasible and safe for a variety of large and hypervascular musculoskeletal tumours. Our small series suggests that preoperative embolisation could contribute to the reduction of the intra-operative and post-operative blood product transfusion. It should be considered as a pre-operative adjunct for major tumour resections with a high risk of bleeding. The use of the haemoglobin gap complemented the assessment of perioperative blood loss.

## Introduction

The management of musculoskeletal tumours is complex and often involves the multiple specialties of orthopaedic surgery, radiology, general surgery, neurosurgery and oncology. Trans-arterial embolisation was first reported by Dr Frieda Feldman in 1975 as a useful adjunct in the management of selective bone tumours^1^. Its role in the treatment of musculoskeletal tumours had become increasingly important with the use of arterial embolisation as a neoadjuvant therapy before surgical resection^2-7^.

The procedure of pre-operative embolisation itself was complex and various techniques and embolic agents had been described^8, 9^. The main aim of pre-operative embolisation was to achieve devascularisation of the tumour before surgical resection, with reduction of intra-operative blood loss and complication rates^10,11^. Early resection was preferred following embolisation, as revascularisation was seen at six weeks in some series^5^.

There are several studies on embolisation of musculoskeletal tumours for neoadjuvant, therapeutic and palliative therapy^12-14^. However, data was limited on the outcomes of preoperative embolisation of musculoskeletal tumours. In this retrospective study, we aimed to report on our experience and add onto the knowledge of safety, technical success and efficacy of pre-operative embolisation in musculoskeletal tumours. In our series, all embolisations were performed with either polyvinyl alcohol (PVA) particles alone or in combination with other embolic agents such as gelfoam slurry or micro coils.

## Materials and Methods

This study was approved by our institutional review board, and the requirement for informed consent was waived. A total of 13 consecutive patients who were planned for pre-operative embolisation from May 2012 to January 2016 were enrolled in the study. The indications for pre-operative embolisation were discussed at a multidisciplinary tumour board, considering tumour size, histology and location, vascularity and estimated blood loss from surgery. Larger tumours, anatomically challenging locations for resection and tumours with hypervascularity and neovascularity on CT angiography were considered and a collective decision made for pre-operative embolisation at the tumour board meeting with the orthopaedic surgeon, interventional radiologist, diagnostic radiologist, medical oncologist and pathologist.

Histological diagnosis of the tumour was confirmed by either open or core needle biopsy before definitive surgery. Location of the tumour was determined by pre-operative imaging with plain radiographs, computed tomography (CT) or magnetic resonance imaging (MRI). Size of the tumour was quantified by measuring the anterior-posterior, craniocaudal and transverse length on CT or MRI scans before embolisation.

All embolisation procedures were performed while the patient was under conscious sedation induced by intravenous fentanyl and midazolam. Vascular access was obtained via the common femoral artery, and a 5F or 6F vascular sheath was used. The appropriate 4F or 5F pre-shaped catheters were used for the diagnostic angiography. All embolisations were done using a micro-catheter system coaxially introduced through the 4F or 5F catheter with selective catheterisation of the tumour feeders. The embolisation procedural endpoint was reached when stasis was observed in the feeding arteries along with the significant reduction in tumour blush. A completion angiogram was performed to evaluate overall devascularisation of the tumour. The musculoskeletal interventional radiologist retrospectively reviewed the images obtained before and after an embolisation. The effect of pre-operative embolisation on obliterating the tumour blush was based on a comparison of the pre- and post-embolisation angiograms on the PACS monitors. The criteria for technical success included catheterisation of the significant afferent tumour-supplying vessels and total interruption of the blood supply to the tumour by 70% or more as in the post-embolisation angiography^4,15^. All procedures were carried out by a single procedurist. The embolic agent was at the discretion of the performing interventional radiologist, with PVA being the preferred embolic agent as it was used for distal tumour microvasculature penetration. Gelfoam was used to occlude proximal medium to larger vessels following deeper embolisation with PVA, reducing the risk of permanent non-target embolisation. Microcoils were similarly used to accurately complete the embolisation following PVA use to avoid recanalisation of the feeding vessel. Occasionally coils were used to embolise and protect non-target vessels originating proximally, which could not be navigated beyond to get closer to tumour feeders, thereby avoiding riskier distal non-target embolisation by PVA.

Operative records were reviewed to determine the period from embolisation to surgery and the type of surgery performed. In our institution, all tumour cases undergoing pre-operative embolisation would have a definitive surgical procedure done within a week. The interval between embolisation and surgery varied and depended on the availability of the embolisation suite and the operating theatre list. Intra-operative blood loss and amount of blood products administered during the surgical procedure by anesthesiologist were recorded. Allogeneic and autologous red blood cells from cell salvage were combined to give the total red blood cells transfused intra-operatively. If the surgery was in two stages, the sum of the intra-operative blood loss (IBL) and the packed red blood cells (PRBC) units transfused for each stage were added together and the total used for the analysis.

Pre, immediate, post-operative and subsequent haemoglobin levels were obtained from the online medical records and any transfusion within the week following surgery was determined from online records of blood products ordered. Descriptive data were presented as medians and means with ranges, if appropriate; categorical data were presented as percentages.

## Results

A total of 13 patients, of which five were females and eight were males, underwent pre-operative embolisation during the study period (Table I). The age ranged from 16 to 68 years, and the median age was 54 years. Metastatic tumours made up 23% (n=3) of this series. Of all the primary tumours (n=10, 77%), one of them was benign (giant cell tumour), the rest being malignant. Location of tumours was varied; with one tumour (8%) in the upper limb, four tumours (31%) in lower limbs, one tumour (8%) in the spine and seven tumours (53%) in the pelvic region.

**Table I:** Tumour characteristics, surgical procedure done, blood loss and blood products given intra-operatively and post-operatively

No	Sex	Age	Tumour diagnosis	Location	Tumour Size AP x CC x TL (cm)	EN	Agent/s	Size (Microns)	CX	DS (%)	Interval (days)	Operation done	IBL (ml)	IBO (ml)	Pre-op Hb (g/dL)	Post-op Hb (g/dL)	Hb gap (pre-op minus post-op)	Subsequent post-op Hb (g/dL)	Post-op transfusion given
1	M	24	Solitary fibrous tumour	Left scapula	7 x 6 x 9.5	1	PVA	355-500	Nil	90	3	Resection	380	Nil	15	12	-3	Not measured	Nil
2	M	39	Spindle cell sarcoma	Left groin	5.5 x 16 x 5.5	2	PVA	355-500	Nil	90	5	Resection	250	PRBC 278, FFP 500	10.8	9.7	-1.1	Not measured	Nil
3	M	58	Leiomyosarcoma	Left thigh	9.5 x 12 x 12	1	PVA	355-500	Nil	90	1	Resection	150	Nil	12.8	10.8	-2	12.2	Nil
4	F	33	Chondrosarcoma	Right iliac	12x9x13	1	PVA	355-500	Nil	90	2	Hemi- pelvectomy	NK	PRBC 55	11.9	7.9	-4	10.5	PRBC 2 units
5	M	20	Osteosarcoma	Left iliac	18 x 18x9	1	PVA	355-500	Nil	80	4	Hemi- pelvectomy	NK	PRBC 470	14.1	9.9	-4.2	9.4	PRBC 2 units
6	F	52	Thyroid carcinoma metastases	Left femur	13x7.5x 7.5	1	PVA	355-500	Nil	80	2	Resection, endoprosthesis	1500	PRBC 300	12.5	7.3	-5.2	9.1 4 units	PRBC
7	F	66	Thyroid carcinoma metastases	Left femur	9.5 x 13 x 9.5	2	PVA	355-500	Nil	85	6	Curettage, Plating	300	PRBC 304	9.1	10.2	+1.1	9.4	Nil
8	F	55	Leiomyosarcoma	Spine T9	8.5 x 6.5 x 8.0	1	PVA, Microcoils	250-355	Nil	90	0	Laminectomy, Excision, Instrumentation	500	Nil	12.6	10.2	-2.4	10	PRBC 1 unit
9	M	64	Metastases	Left hemi- pelvis	10 x 9.5 x 18	1	PVA	150-250	Nil	80	3	Excision	1000	PRBC 617, FFP 505	10.7	11.1	+0.4	12.1	Nil
10	F	16	Giant cell tumour	Right hemi- pelvis	10x9.5x 18	1	PVA, Gelform	355-500	Nil	100	7	Curettage	250	PRBC 568	12.1	12.5	+0.4	Not measured	Nil
11	M	59	Chordoma	Sacrum	10x9.5x 18	1	PVA, Gelform	250-355	Nil	90	6	Resection, Hartmann's procedure	500	Nil	12.3	9.9	-2.4	7.2	PRBC 3 units, FFP 1 unit
12	M	54	Chordoma	Sacrum	15.5x15.5x 21	1	PVA, Microcoils	355-500	Nil	90	4	Resection, Abdominal Perineal Resection	3700	NK	10	15.3	+5.3	12.3	PRBC 3 units, FFP 2 units, HAS 1 unit
13	M	68	Chordoma	Sacrum	12 x 12x 12	1	PVA, Gelform	355-500	Nil	90	7	Wide Resection Abdominal perinea Resection, Internal iliac ligation	6900	PRBC 4921	13.4	8.6	-4.8	10.5	PRBC 2 units

EN = Number of embolisation, CX = Complications during embolisation, PVA = Polyvinyl alcohol particles, DS = devascularisation status, Interval = Time between last embolisation and operation, IBL = Intraoperative Blood Loss, IBP = Intraoperative Blood Products given, PRBC = Packed Red Blood Cells, FFP = Fresh Frozen Plasma, NK = Not known, PRBC 1 unit = 300ml of packed red blood cells, FFP 1 unit = 250ml of Fresh Frozen Plasma, HAS 1 unit = 100ml of Human Albumin Solution

All embolisations were performed with either PVA alone or in combination with other embolic agents such as gel foam slurry or micro coils (Table I). The size of PVA used varied within the series with the majority (69%), being medium-sized of 355 to 500 microns, to achieve adequate devascularisation. Two cases underwent two-stage embolisation for adequate devascularisation, as they were for hypervascular tumours; a metastatic thyroid carcinoma and a spindle cell sarcoma. The technical success rate was 100% as all tumours were devascularised more than 70%. Of the 15 embolisation procedures (n=13), one (7%) dissection occurred during attempts to cannulate a tortuous feeding artery from the anterior division of the internal iliac artery. The remaining feeding vessels from the internal iliac artery were successfully cannulated with successful devascularisation of the tumour. The mean interval between the last embolisation and surgical intervention was 3.8 days (range 0-7 days).

The majority of the surgical interventions were either a resection or excision of the tumour in nine patients (n=9, 69%). Two cases underwent internal hemipelvectomy (15%) and two had a curettage (15%). There were two patients with thyroid metastases, Cases 6 and 7; one patient had an endoprosthesis inserted after the resection of the left proximal femur and the other patient had the diaphysis of the femur plated after a curettage of the tumour. A patient with a T9 spinal metastasis, Case 8, required a laminectomy with further posterior instrumentation of T7-11 vertebrae. Case 10 was a patient with a large giant cell tumour of the hemipelvis and required a curettage of the lesion, with subsequent monthly Denosumab as an adjuvant therapy with the medical oncologist. Of the three patients with sacral chordoma, two required an abdominal perineal resection as the tumour had extended into the lower rectum, another patient required a concurrent Hartmann’s procedure as the tumour had extended into the recto-sigmoid junction. One of the 3 patients, case number 13, required internal iliac vessels mobilisation and ligation as the tumour was found to be encasing the vessels; this resulted in significant blood loss (6900mls).

Mean intra-operative blood loss was 1403ml with a range of 150ml to 6900ml, in nine patients (n=11). Eight patients (n=13, 62%) required intra-operative packed red blood cells (PRBC). The mean volume of PRBC given during surgery was 939ml (55ml - 4921ml) for the eight patients who had intra-operative blood products. Seven patients (54%) required further transfusion post-operatively due to persistent low haemoglobin. In the group of patients who received an intra-operative transfusion, four patients went on to receive further transfusion post-operatively. The mean post-operative blood transfusion units required by patients was 2.1 units.

Haemoglobin parameters are summarised in Table I. The mean haemoglobin level was 12.1g/dL pre-operatively and 10.4g/dL post-operatively. The mean drop in haemoglobin after the surgical intervention was calculated to be 1.7g/dL.

## Discussion

**Fig. 1:**
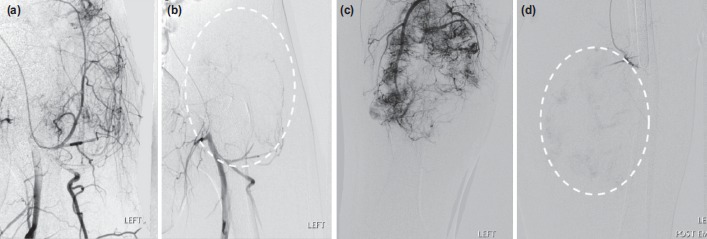
(a,b) A 39-year-old gentleman presented with spindle cell sarcoma in the left groin and pre-operative angiography was performed. The pre-embolisation image demonstrates tumour neo-vascularity from the left femoral artery. The feeding arteries were embolised with 355-500um PVA particles. The post embolisation image demonstrated more than 90% devascularisation. (c, d) A 65-year-old female patient with metastatic follicular cell thyroid carcinoma involving left femur and adjacent soft tissues. The pre-embolisation image demonstrates neo-vascularity with feeders from superficial and profunda femoral arteries. The feeders were embolised with 355-500 um PVA particles. The post embolisation image demonstrated successful devascularisation.

In our series, pre-operative embolisation was successfully performed in all patients following the criteria of technical success with the catheterisation of the tumour-supplying arteries and the obliteration of >70% of the tumour staining mass^4,15-18^. Selected case examples of successful embolisation are shown in Fig. 1.

Studies have shown that successful embolisation of musculoskeletal tumours could reduce intra-operative blood loss^15,19^, improve visualisation intra-operatively and thus completeness of the resection margins. This was particularly important in large tumours in which challenging en-block resections were contemplated, with risks of life-threatening haemorrhage^6^. In a large series of 51 patients, Barton *et al*^16^ reported intra-operative blood loss of 500 - 1,500ml in patients who had undergone pre-operative embolisation of bone tumours; patients who had not undergone any embolotherapy had an intra-operative blood loss of 2,000 - 18,500ml. Manke *et al*^20^ reported significantly reduced intra-operative haemorrhage in patients with spinal metastases from renal cancer, following pre-operative embolisation with PVA particles compared to patients treated surgically without pre-operative embolisation. Kickuth *et al*^21^ reported a median intra-operative blood loss of 600ml with a range of 200 – 4,000ml for patients who have had undergone pre-operative devascularisation of bone tumours. Earlier studies on preoperative embolisation of musculoskeletal tumours had demonstrated its efficacy in reducing intra-operative blood loss and these results provided a baseline for comparison.

In our study, we found our intra-operative blood loss to range from 150ml to 6,900ml with a mean of 1173ml and a median of 500ml, which was comparable to the literature. The mean blood loss calculated in this series was skewed by Case number 13, with a sacral chordoma resection where intra-operative blood loss was noted to be at 6900ml.

There were two patients Case number 4 and 5, where intra-operative blood losses were not documented. Blood losses in these two cases were then approximated from the change in haemoglobin post-operatively which were -4 and -4.2 for patient number 4 and 5, respectively. These suggested a significant but not an extensive amount of blood loss. Coupled with a minimal amount of blood products transfused post-operatively, we could then gauge the intra-operative blood loss. In Case number 12 where the intra-operative blood products given were not known, we could postulate that significant blood product was given as there was a large positive increase in the haemoglobin gap (+5.3) post-operatively. In our case series, the use of the hemoglobin gap serves as a useful adjunct to assess intra-operative blood loss as well as blood products given. The use of haemoglobin gap in postulating blood loss/blood products given was also supported by Bapuraj *et al’s* case-control study^22^ where the group of patients with pre-operative embolisation had a significantly lower haemoglobin drop post-operatively compared to the control group. Some authors also believe that estimated blood loss, being a semiquantitative variable with a potential source of bias, could sometimes be inaccurate^23^. Hence, the haemoglobin gap could provide a clearer peri-operative blood loss profile.

Post-operative transfusion requirement beyond the immediate peri-operative period was not often documented in published embolisation studies. In our series, postoperative transfusion requirement within one week of postoperative period averaged 2.1 units. The indication for transfusion beyond the immediate peri-operative period was based on clinical evaluation of the post-operative haemoglobin trend, symptoms related to anaemia as well as surgical drain output. No patient developed massive postoperative haemorrhage with hemodynamic instability requiring escalation of care, ionotropic support or further intervention such as angioembolisation or surgical exploration. Our case series suggests that a combination of pre-operative musculoskeletal tumour embolisation with major surgery could reduce the risk of severe delayed secondary haemorrhage, despite the many potential confounding factors including surgical technique, tumour type and location, and even concurrent resection of bowel.

Apart from reducing peri-operative bleeding, embolisation for musculoskeletal tumours could also reduce the amount of viable tumour, by inhibiting tumour growth and decreasing required doses of radiotherapy and chemotherapy. It could also be performed as a stand-alone in the palliative setting for pain relief^21, 24^ and reducing tumour volume^25^ or combined with ablation and cementoplasty^9^. Major complications arising from pre-operative tumour embolisation were uncommon^12, 15, 16, 21, 26^ with a non-target embolisation and the post embolisation syndrome being the most frequently encountered^27, 28^. We experienced a small tumour-feeding artery dissection, supplying 5% - 10% of the tumour, arising from the anterior branch of the internal iliac artery during cannulation attempts in Case number 9. Technical success was achieved in that patient and there was no requirement for post-procedure blood transfusion. No other significant complications relating to the pre-operative embolisation were recorded in our retrospective case series. The overall complication rate from pre-operative embolisation was 7% (n=1) which was encouraging compared to the other studies in literature where the overall complication rate ranges from 3.1% to 28.6%^21, 29, 30^.

There were several limitations to our study. Firstly, data collection was retrospective, and comparison with a non-embolisation group was not available. However, by cross-referencing with multiple studies from the literature, we aimed to provide a fair depiction of pre-operative embolisation as an adjunct to orthopaedic tumour surgery. In our series, the median blood loss calculated at 500ml is comparable, if not lower than that was reported in most studies^20, 21, 31, 32^. Secondly, our study population was not homogeneous with regards to tumour types and the type of surgery performed. However, this reflected the clinical setting of primary and metastatic tumours, both referred simultaneously to the interventional radiologists for preoperative embolisations and subsequent operation by a single musculoskeletal tumour surgeon. A prospective randomised trial would be beneficial for defining the exact value of preoperative embolisation compared with surgery without preoperative embolisation. Such a trial would be difficult to design and not likely to be feasible.

## Conclusion

Pre-operative embolisation for musculoskeletal tumours is feasible and safe before surgical intervention for a variety of large and hypervascular musculoskeletal tumours. Our small series suggests that pre-operative embolisation could also contribute to reducing the intra-operative as well as postoperative blood product transfusion requirement and should be considered as an adjunct for major resections with a high risk of bleeding. The use of haemoglobin gap complemented an assessment of peri-operative blood loss.
